# Author Correction: PDX1^LOW^ MAFA^LOW^ β-cells contribute to islet function and insulin release

**DOI:** 10.1038/s41467-021-24848-5

**Published:** 2021-07-20

**Authors:** Daniela Nasteska, Nicholas H. F. Fine, Fiona B. Ashford, Federica Cuozzo, Katrina Viloria, Gabrielle Smith, Aisha Dahir, Peter W. J. Dawson, Yu-Chiang Lai, Aimée Bastidas-Ponce, Mostafa Bakhti, Guy A. Rutter, Remi Fiancette, Rita Nano, Lorenzo Piemonti, Heiko Lickert, Qiao Zhou, Ildem Akerman, David J. Hodson

**Affiliations:** 1 Institute of Metabolism and Systems Research (IMSR), University of Birmingham, Birmingham, UK; 2 Centre of Membrane Proteins and Receptors (COMPARE), University of Birmingham and University of Nottingham, Midlands, UK; 3 Centre for Endocrinology, Diabetes and Metabolism, Birmingham Health Partners, Birmingham, UK; 4 School of Sport, Exercise and Rehabilitation Science, University of Birmingham, Edgbaston, UK; 5 MRC-Versus Arthritis Centre for Musculoskeletal Ageing Research, University of Birmingham, Edgbaston, UK; 6 Institute of Diabetes and Regeneration Research, Helmholtz Zentrum München, D-85764 Neuherberg, Germany; 7 German Center for Diabetes Research (DZD), D-85764 Neuherberg, Germany; 8 Institute of Stem Cell Research, Helmholtz Zentrum München, D-85764 Neuherberg, Germany; 9 Technical University of Munich, School of Medicine, Munich, Germany; 10 Section of Cell Biology and Functional Genomics, Division of Diabetes, Endocrinology, and Metabolism, Department of Metabolism, Reproduction, and Digestion, Imperial College London, London, UK; 11 Lee Kong Chian School of Medicine, Nanyang Technological University, Nanyang, Singapore; 12 Institute of Immunology & Immunotherapy, College of Medical and Dental Sciences, University of Birmingham, Birmingham, UK; 13 San Raffaele Diabetes Research Institute, IRCCS Ospedale, San Raffaele, Italy; 14 Vita-Salute San Raffaele University, Milan, Italy; 15 Division of Regenerative Medicine, Department of Medicine, Weill Cornell Medical College, New York, NY USA

**Keywords:** Cell signalling, Type 2 diabetes

Correction to: *Nature Communications* 10.1038/s41467-020-20632-z, published online 29 January 2021.

The original version of this Article contained an error in ref. 45, which was incorrectly given with the wrong author name as: Dzeja, P. et al. Mice deficient in the respiratory chain gene Cox6a2 are protected against high-fat diet-induced obesity and insulin resistance. *PLoS ONE* **8**, e56719 (2013). The correct form of ref. 45 is: Quintens, R. et al. Mice deficient in the respiratory chain gene Cox6a2 are protected against high-fat diet-induced obesity and insulin resistance. *PLoS ONE* **8**, e56719 (2013). This has been corrected in the PDF and HTML versions of the Article.

