# Supplementary material for: PDX1LOW MAFALOW β-cells contribute to islet function and insulin release
Source: Nat Commun. 2021 Jan 29;12:674. doi: 10.1038/s41467-020-20632-z (PMC7846747; doi:10.1038/s41467-020-20632-z)
Supplement: Supplementary file 1 — Supplementary Information [file 41467_2020_20632_MOESM1_ESM.pdf]

SUPPLEMENTARY INFORMATION

PDX1^{LOW} MAFA^{LOW} β-cells contribute to islet function and insulin release

Daniela Nasteska[1-3], Nicholas H.F. Fine[1-3], Fiona B. Ashford[1-3], Federica Cuozzo[1-3], Katrina Viloria[1-3], Gabrielle Smith[1,3], Aisha Dahir[1,3], Peter W.J. Dawson[4,5], Yu-Chiang Lai[1,4,5], Aimée Bastidas-Ponce[6-9], Mostafa Bakhti[6-8], Guy A. Rutter[10,11], Remi Fiancette[12], Rita Nano[13,14], Lorenzo Piemonti[13,14], Heiko Lickert[6-9], Qiao Zhou[15], Ildem Akerman[1,3], David J. Hodson[1-3*]

[1]Institute of Metabolism and Systems Research (IMSR), University of Birmingham, Birmingham, UK
[2]Centre of Membrane Proteins and Receptors (COMPARE), University of Birmingham and University of Nottingham, Midlands, UK.
[3]Centre for Endocrinology, Diabetes and Metabolism, Birmingham Health Partners, Birmingham, UK
[4]School of Sport, Exercise and Rehabilitation Science, University of Birmingham, UK
[5]MRC-Versus Arthritis Centre for Musculoskeletal Ageing Research, University of Birmingham, Edgbaston, UK
[6]Institute of Diabetes and Regeneration Research, Helmholtz Zentrum München, D-85764 Neuherberg, Germany
[7]German Center for Diabetes Research (DZD), D-85764 Neuherberg, Germany
[8]Institute of Stem Cell Research, Helmholtz Zentrum München, D-85764 Neuherberg, Germany
[9]Technical University of Munich, School of Medicine, Munich, Germany
[10]Section of Cell Biology and Functional Genomics, Division of Diabetes, Endocrinology, and Metabolism, Department of Metabolism, Reproduction, and Digestion, Imperial College London, London, UK
[11]Lee Kong Chian School of Medicine, Nanyang Technological University, Singapore
[12]Institute of Immunology & Immunotherapy, College of Medical and Dental Sciences, University of Birmingham, B15 2TT, Birmingham, UK
[13]San Raffaele Diabetes Research Institute, IRCCS Ospedale San Raffaele, Italy
[14]Vita-Salute San Raffaele University, Milan, Italy
[15]Division of Regenerative Medicine, Department of Medicine, Weill Cornell Medical College, New York, USA

*Corresponding author: David J. Hodson, d.hodson@bham.ac.uk

SUPPLEMENTARY FIGURES

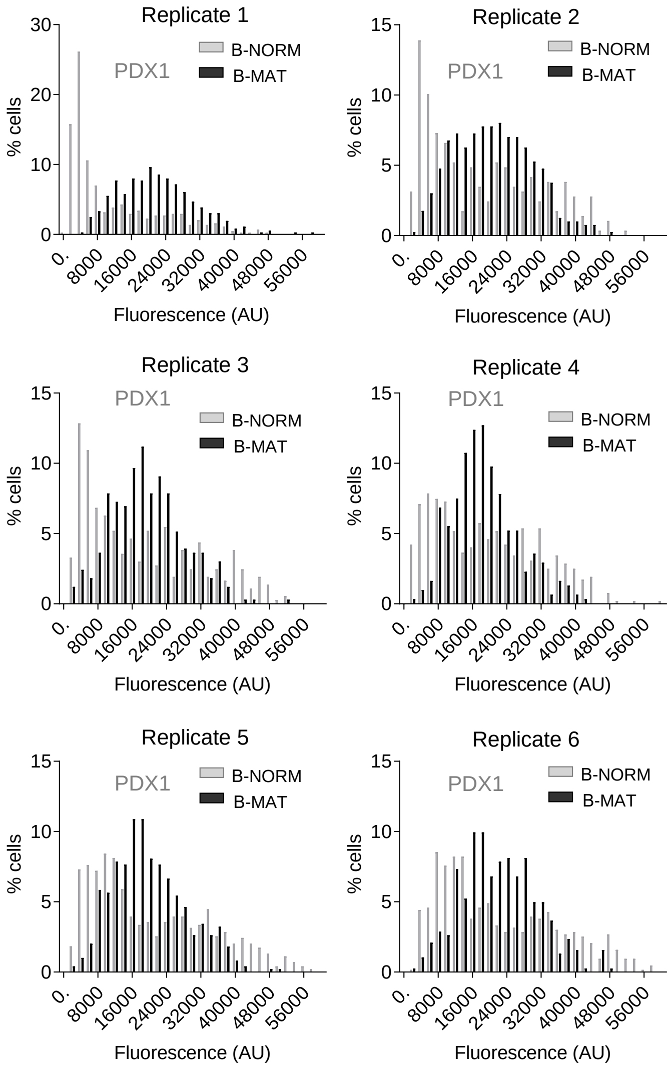

Supplementary Figure 1: PDX1 fluorescence intensity distribution plots. PDX1 fluorescence intensity distribution plots shown by individual replicate (n = 6).

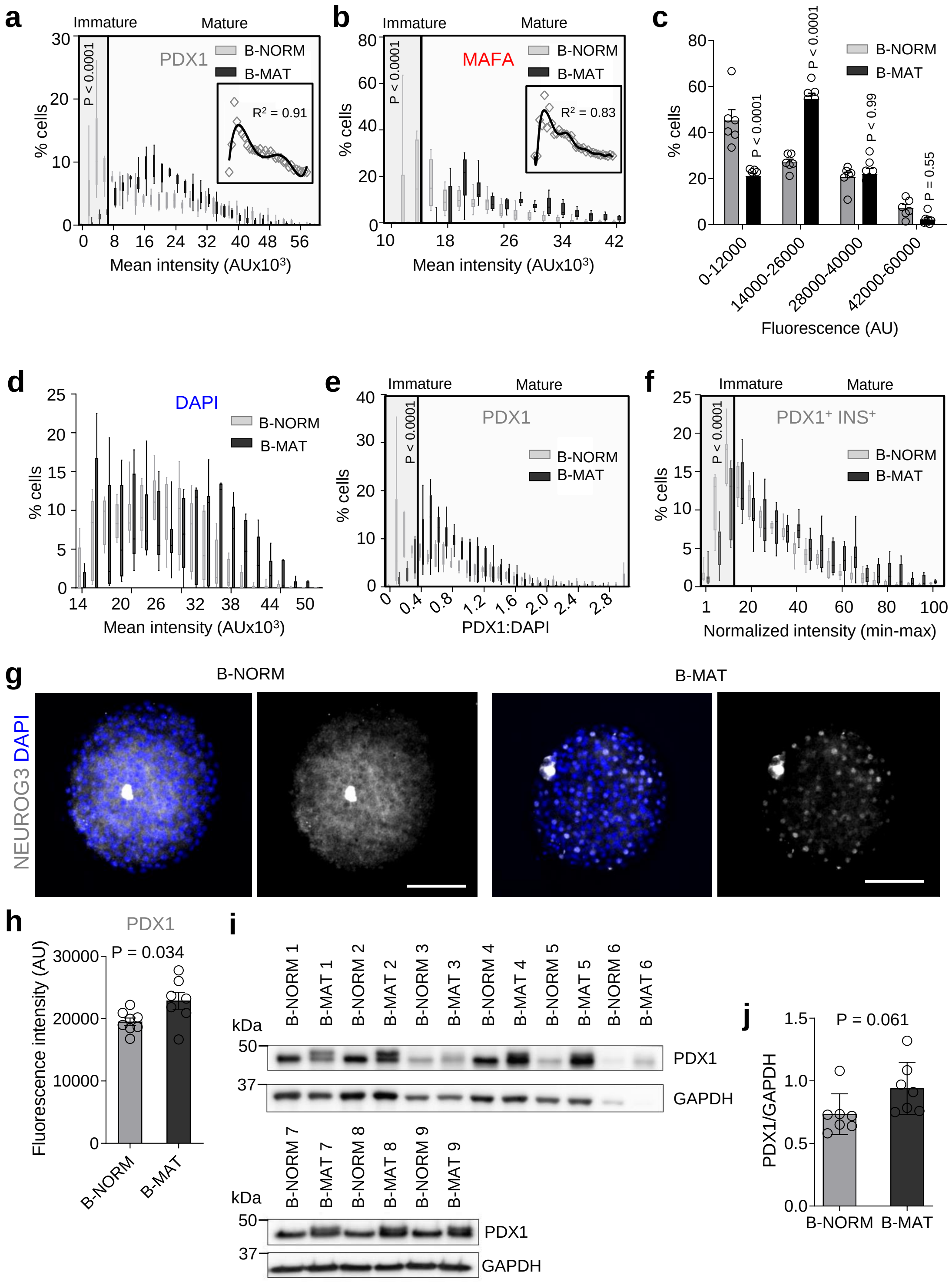

Supplementary Figure 2: Raw data and normalization controls for mouse B-MAT islets, NEUROG3 expression and overexpression strength. a, b Non-normalized PDX1 (**a**) and MAFA (**b**) fluorescence intensity-probability distribution showing a decrease in the proportion of cells occupying the bottom 15 percentile for PDX1 and MAFA expression in B-MAT versus B-NORM islets (n = 6 islets/3 animals; two-way ANOVA, Bonferroni's multiple comparison) (PDX1: F = 13.38, DF = 30) (MAFA: F = 3.98, DF = 32). **c**, As for **a**, but wider bins showing no changes in the proportion of cells occupying the higher fluorescence intensity ranges in B-MAT versus B-NORM islets (n = 6 islets/3 animals; two-way ANOVA, Bonferroni's multiple comparison)) (F = 43.25, DF = 3). **d** DAPI nuclear staining intensity distribution is similar in normal (β-cell normal; B-NORM) and Ad-M3C-transduced (β-cell

mature; B-MAT) islets (n = 6 islets/3 animals; two-way ANOVA). **e** Loss of immature β-cells is still evident in B-MAT islets following normalization of PDX1 expression levels versus DAPI for each cell analyzed (n = 6 islets/3 animals; two-way ANOVA) (F = 4.9, DF = 30). **f** As for **e**, but taking into account only cells that are positive for both insulin (INS) and PDX1 (n = 6 islets/3 animals; two-way ANOVA) (F = 4.1, DF = 20)**. g** NEUROG3 cannot be detected in normal B-NORM islets using specific antiserum, but is faintly present in B-MAT islets (scale bar = 85 µm). **h** Whole-islet PDX1 fluorescence intensity is only slightly increased in B-MAT versus B-NORM islets (n = 7 islets/3 animals; unpaired t-test). **i** Western blot showing PDX1 and GAPDH expression (run in parallel on separate blots) in B-NORM and B-MAT islets (n = 9 animals, labelled 1 through 9; samples 1 -6 and 7-9 were run on separate gels processed in parallel). A slight shift in the PDX1 band in B-MAT islets is apparent due to the presence of exogenous PDX1. **j** Densitometric quantification of the blots shown in (**i**) (n = 8 animals, labelled 1-5 and 7-9). Note that B-NORM 6 and B-MAT 6 were omitted from analysis due to poor abundance (i.e. GAPDH was undetectable). Bar graphs show the mean ± SEM. Box-and-whiskers plot shows median and min-max. All tests are two-sided where relevant.

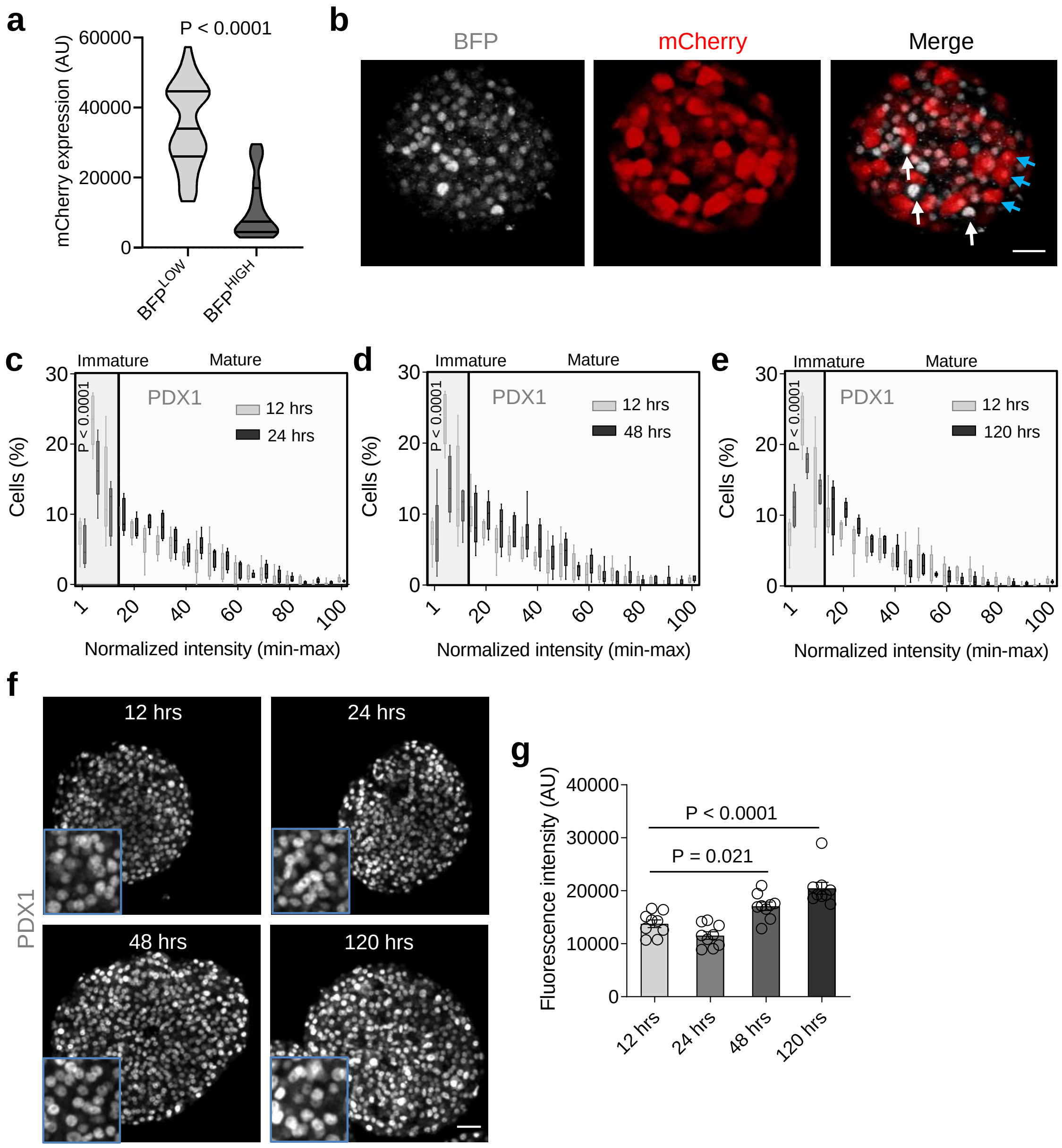

Supplementary Figure 3: Overexpression localization and time course studies for Ad-M3C. a mCherry expression and thus Ad-M3C viral transduction is significantly higher in BFP^{LOW} cells (i.e. immature) (n = 48 cells from 7 islets) (unpaired t-test). **b** Representative images showing mCherry expression in Pdx1-BFP islets 48 hours following infection with Ad-M3C. White arrows show BFP/PDX1^{HIGH} cells with low mCherry levels, whereas blue arrows show BFP/PDX1^{LOW} cells with high mCherry levels (scale bar = 26.5 µm). **c-e** The proportion of β-cells occupying the bottom 15 percentile for PDX1 expression (PDX1^{LOW}) is reduced in islets transduced with Ad-M3C at 24 hrs post-infection (**c**), and this change is sustained at 48 hrs (**d**) and 120 hrs (**e**) post-infection (n = 6 islets/3 animals; two-way ANOVA, Bonferroni's multiple correction) (24 hrs: F = 3.01, DF = 20) (48 hrs: F = 2.24, DF = 20) (120 hrs: F = 4.9, DF = 20). Note that the same 12 hrs (control) PDX1 fluorescence intensity distribution is shown in all graphs to allow cross-comparison (the experiments were performed in parallel). **f** Representative images showing PDX1 staining at 12-120 hrs post-infection (scale bar = 25 µm). **g** An increase in PDX1 fluorescence is apparent after 48 hrs, as overexpression continues to increase in the targeted cells (n = 9 islets/3 animals; one-

way ANOVA, Sidak's multiple correction) ($F = 20.83$, $DF = 3$). Bar graphs show the mean ± SEM. Violin plot shows median and interquartile range. Box-and-whiskers plot shows median and min-max. All tests are two-sided where relevant.

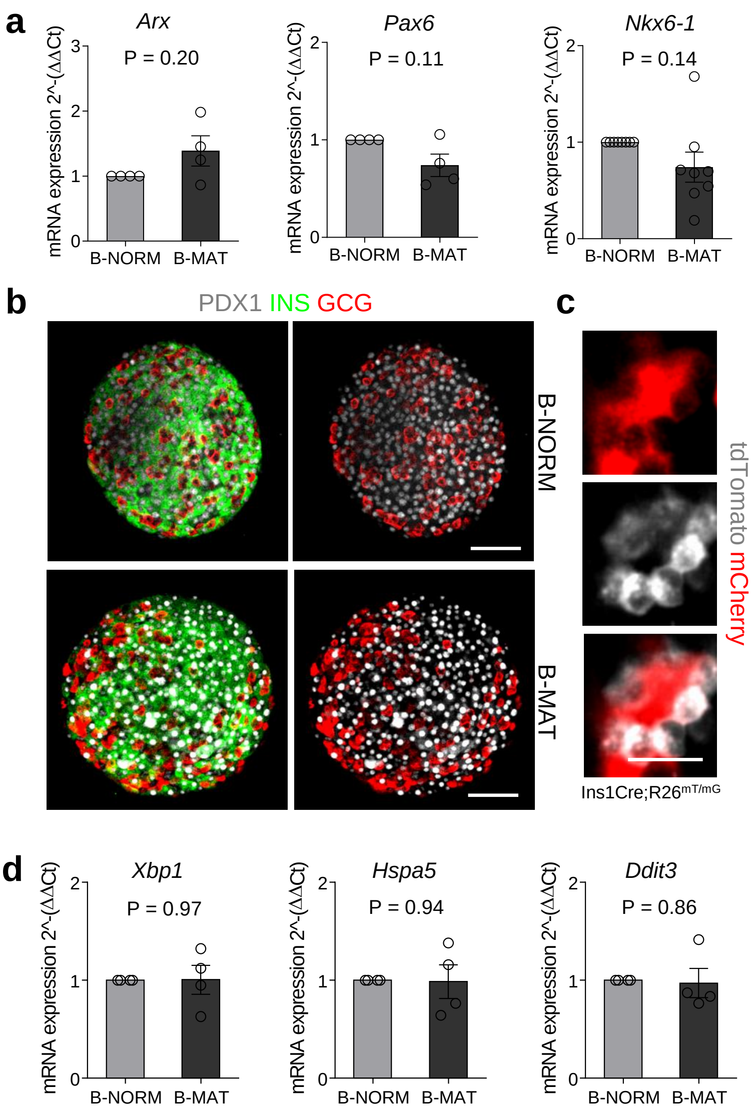

Supplementary Figure 4: Overexpression is largely confined to β-cells and ER stress markers are not upregulated. **a** Expression levels of *Arx, Pax6* and *Nkx6-1* are not significantly altered in B-MAT islets (n = 4 animals; paired t-test). **b** No difference in the proportion of PDX1$^+$GCG$^+$ cells was detected in B-NORM compared to B-MAT islets (scale bar = 120 µm). **c** Imaging of live Ins1Cre;R26mT/mG reporter islets was used to preserve mCherry expression for assessment of localization. No mCherry expression was detected in non- β-cells (tdTomato; grey) at the viral titres used here (n = 17 islets/2 animals) (scale bar = 25 µm). **d** Expression of the ER stress/UPR markers *Xbp1*, *Hspa5* and *Ddit3* is not significantly altered in B-MAT islets (n = 4 animals; paired t-test). Bar graphs show the mean ± SEM. All tests are two-sided where relevant. ER-endoplasmic reticulum; UPR-unfolded protein response.

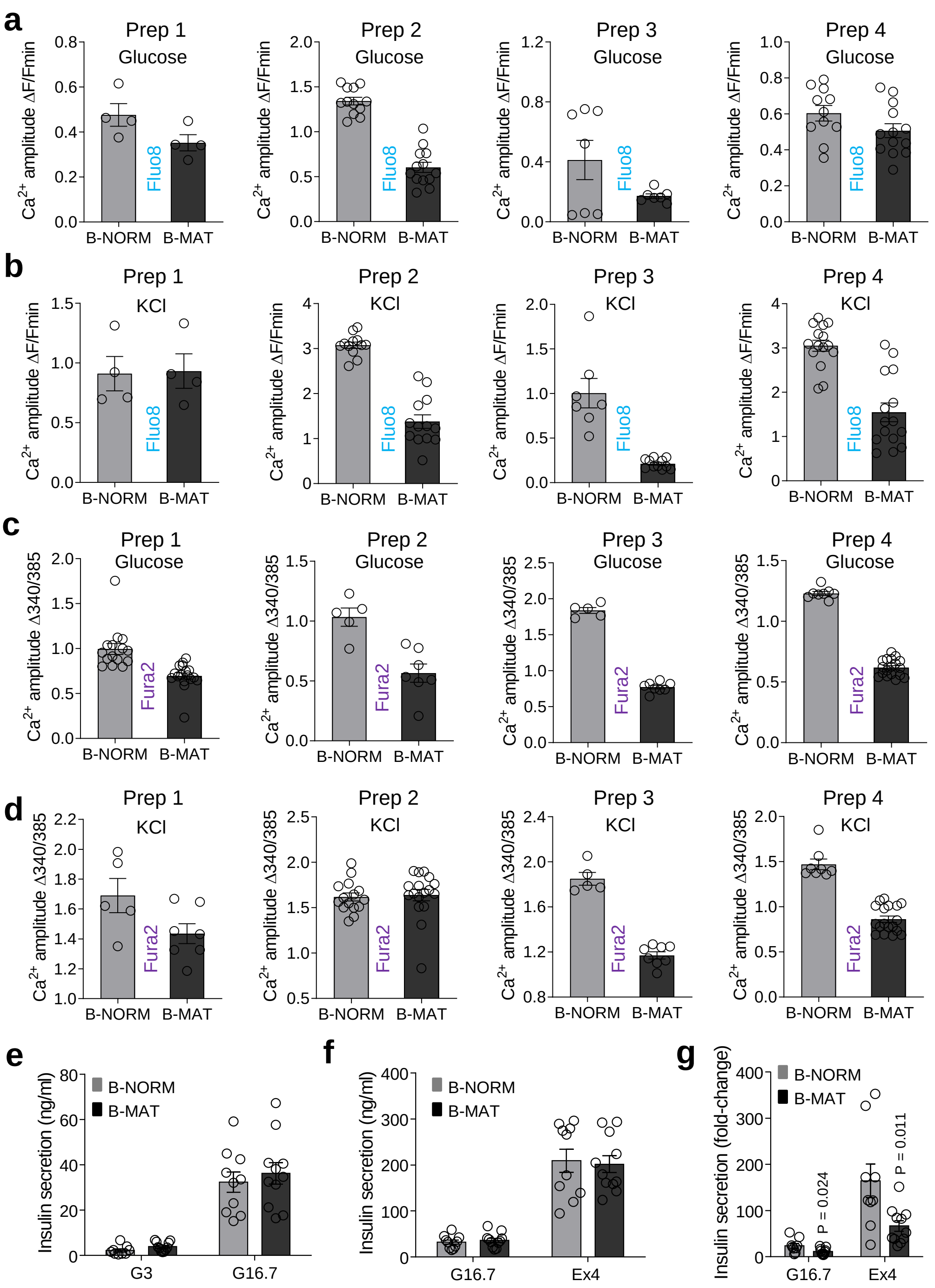

Supplementary Figure 5: Ca^{2+} recordings shown by individual preparation, raw insulin secretion data and fold-change insulin release. a, b Summary bar graphs showing amplitude of Ca^{2+} responses to glucose (**a**) or KCl (**b**) for each separate islet isolation/preparation, measured using Fluo8 (n = 34 islets/4 preparations/4-5 animals). **c**, **d** Summary bar graphs showing amplitude of Ca^{2+} responses to glucose (**c**) or KCl (**d**) for each separate islet isolation/preparation, measured using Fura2 (n = 33 islets/4 preparations/4 animals). **e** Uncorrected basal and glucose-stimulated insulin secretion in B-NORM and B-MAT islets (n = 8 replicates/4 animals). **f** As for **e**, but raw Exendin-4-stimulated insulin secretion (n = 8 replicates/4 animals). **g** As for **e** and **f**, but fold-change glucose- and Exendin-4-stimulated insulin secretion in B-NORM and B-MAT islets (n = 8 replicates/4 animals; paired t-test). Note that all samples were run together with the same low glucose control, but due to the relative magnitude, Exendin-4 responses are displayed separately

with the same high glucose state (G3, 3 mM glucose; G16.7, 16.7 mM glucose; Ex4, 20 nM Exendin-4). Bar graphs show the mean ± SEM. All tests are two-sided where relevant.

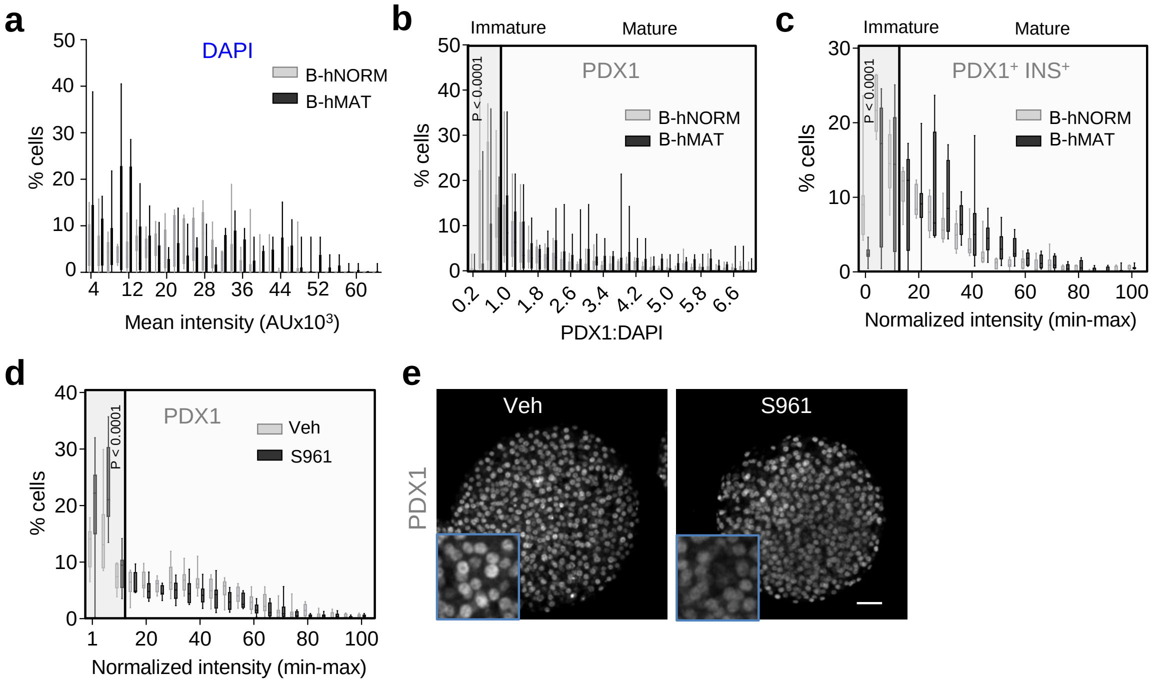

Supplementary Figure 6: Normalization controls for B-hMAT islets, and insulin receptor antagonist experiments. **a** DAPI nuclear staining intensity distribution is similar in normal (β-cell normal; B-hNORM) and Ad-M3C-transduced (β-cell mature; B-hMAT) human islets (n = 14 islets/4 donors; two-way ANOVA). **b** Loss of immature PDX^{LOW} β-cells is still evident in B-hMAT islets following normalization of PDX1 expression levels versus DAPI for each cell analyzed (n = 13 islets/4 donors; two-way ANOVA, Bonferroni's multiple comparison) (F = 2.32, DF = 34). **c** As for **b**, but taking into account only cells that are positive for both insulin (INS) and PDX1 (n = 8 islets; two-way ANOVA, Bonferroni's multiple comparison) (F = 6.15, DF = 20)**. d, e** Treatment with the insulin receptor antagonist, S961 50 nM, increases the proportion of immature PDX1^{LOW} β-cells in wild-type islets versus vehicle (Veh)-treated controls, as shown by frequency distribution (**d**) (F = 3.97, DF = 20) and representative images (**e**) (scale bar = 25 µm) (n = 8 islets/3 animals; two-way ANOVA, Bonferroni's multiple comparison). Box-and-whiskers plot shows median and min-max. All tests are two-sided where relevant.

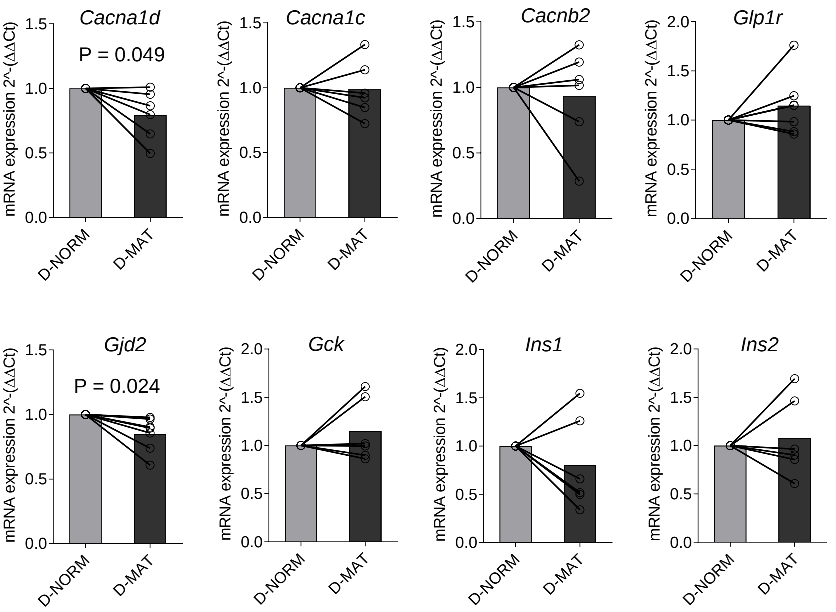

Supplementary Figure 7: Gene expression analyses in D-NORM and D-MAT islets. mRNA levels for *Cacna1*d, *Cacna1c*, *Cacnb2*, *Glp1r*, *Gjd2*, *Gck*, *Ins1* and *Ins2* in D-NORM versus D-MAT chemogenetic islets (n = 6 animals; paired t-test). Bar graphs show paired observations (before and after). All tests are two-sided where relevant.

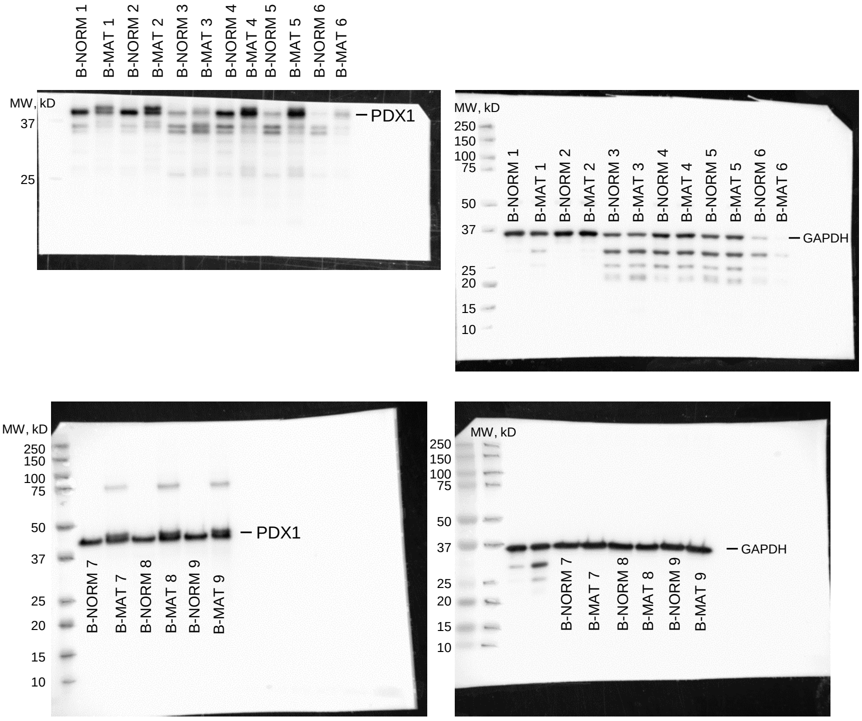

Supplementary Figure 8: Full blot scans corresponding to Supplementary Figure 2i.

SUPPLEMENTARY TABLES

Unique Identifier	Age group (years)	Gender	BMI (kg/m^2)	Glycemia (mmol/L)* HbA1C (%)	History of diabetes **	Islet purity (%)	Islet culture duration (h)	Country of origin
HP1267	50-55	♂	26.5	6.4 mmol/L	No	90	16	Italy
HP1300	20-25	♂	23.5	5.6 mmol/L	No	70	18	Italy
HP1301	60-65	♀	29	11.6 mmol/L	No	80	14	Italy
HP1306	45-50	♂	27.7	6.8 mmol/L	No	80	18	Italy
HP1319	60-65	♂	23.1	5.2 mmol/L	No	90	42	Italy
HP1325	60-65	♀	23.9	6.1 mmol/L	No	60	19	Italy
HP1331	60-65	♀	19.5	7.1 mmol/L	No	90	16	Italy
HP1332	60-65	♀	29.3	6.1 mmol/L	No	90	14	Italy
HP1356	50-55	♀	19.3	7.1 mmol/L	No	80	16	Italy
HP1365	60-65	♀	27.7	9.3 mmol/L	No	70	65	Italy
HP1373	65-70	♀	21.5	7.1 mmol/L	No	65	18	Italy
R277	45-50	♂	33.7	5.6%	No	75	114	Canada
R278	55-60	♂	27.6	5.7%	No	80	17	Canada
R292	45-50	♂	27.6	5.6%	No	90	17	Canada
R330	35-40	♀	24.2	5.1 %	No	85	33	Canada
R340	35-40	♂	23.3	5.3%	No	95	16	Canada
R341	40-45	♂	30	n/a	No	95	34	Canada
R344	55-60	♂	27.8	5.5%	No	95	42	Canada

Supplementary Table 1: Human islet donor characteristics. *: glycemia values during Intensive Care Unit stay **: not diagnosed ante-mortem; ♂-male; ♀-female; n/a-not available; ages are reported as a range in line with the journal indirect identifier policy.

**Primers**		
**SYBR Green primers**		
β-actin	Sigma	For: CGAGTCGCGTCCACCC<br>Rev: CATCCATGGCGAACTGGTG
Pdx1-native	Sigma	For: ACTTAACCTAGGCGTCGCACAAGA<br>Rev: GGCATCAGAAGCAGCCTCAAAGTT
Pdx1-viral	Sigma	For: TGCCTTCGGGCCTTAGCGT<br>Rev: CCGGGATTCTCCTCCACGT
MafA-native	Sigma	For: CGGGAACGGTGATTGCTTAG<br>Rev: GGAGGTTGGGACGCAGAA
MafA-viral	Sigma	For: TTCCCTCGGGAGCCCTCG<br>Rev: TCTCAACATCTCCAGCCAA
Neurog3-native	Sigma	For: AGGTGATCTGCCTTCTTCTGCACT<br>Rev: ACCGTCCCTGCAACTCACACTTTA
Neurog3-viral	Sigma	For: GCTCCCCATCCTATCTGC<br>Rev: TCTTCAACATCTCCTGCTT
Gck	Sigma	For: AGCTGCACCCGAGCTTCA<br>Rev: GATTTCGCAGTTGGGTGTCA
Arx	Sigma	For: TTCCAGAAGACGCACTACCC<br>Rev: TCTGTCAGGTCCAGCCTCAT’
Pax6	Sigma	For: CAGTGTCTACCAGCCAATCC<br>Rev: GCACTGTACGTGTTGGTGAG
Nkx 61	Sigma	For: GCCTGTACCCCCCATCAAG<br>Rev: GTGGGTCTGGTGTGTTTTCTCTT
Cacna1c	Sigma	For: CCAACCTCATCCTCTTCTTCA<br>Rev: ACATAGTCTGCATTGCCTAGGAT
Cacna1d	Sigma	For: GAAGCTGCTTGACCAAGTTGT<br>Rev: AACTTCCCCACGGTTACCTC
Cacnb2	Sigma	For: GCAGGAGAGCCAGATGGA<br>Rev: TCCTGGCTCCTTTTCCATAG
Rfx6	Sigma	For: TGCCAGTGCATACTCGACAAT<br>Rev: AACAGGATTTTCAAGCAGGGG
Xbp1	Sigma	For: AGCAGCAAGTGGTGGATTTG<br>Rev: GAGTTTTCTCCCGTAAAAGCTGA
Hspa5	Sigma	For: ACTTGGGGACCACCTATTCCT<br>Rev: GTTGCCCTGATCGTTGGCTA
Ddit3	Sigma	For: CTGGAAGCCTGGTATGAGGAT<br>Rev: CAGGGTCAAGAGTAGTGAAGGT
Glp1r	Sigma	For:<br>GGGTCTCTGGCTACATAAGGACAAC<br>Rev: AAGGATGGCTGAAGCGATGAC
Adcy8	Sigma	For: TTGGGCTTCCTACACCTTGACT<br>Rev: CGGTAGCTGTATCCTCCATTGAG
Gjd2	Sigma	For: GATTGGGAGGATCCTGTTGAC<br>Rev: AGGGCTAGGAAGACAGTAGAG
Ins1	Sigma	For: GTCGGTGGGCATCCAGTAA<br>Rev: AATGACCTGCTTGCTGATGGT
Ins2	Sigma	For: GAAGTGGAGGACCCACAAGT<br>Rev: GATCTACAATGCCACGCTTC
Cox6a2	Sigma	For: GCCCAGCAAGATTCTGTGATG<br>Rev: TCTGGATGTCGGGTAAGGCAT
G6pc2	Sigma	For: ACTCCACAGAAAGGACCAGG<br>Rev: GTCATGGTAACAGCTGCCCT
Ascl1	Sigma	For: TCGTTGGCGAGAAACACTAA<br>Rev: AGGAACAAGAGCTGCTGGAC
Ero1LB	Sigma	For: TGCTGTCAATGTCACATAAGC<br>Rev: AACTGCTTGTCACCCTGAGC
Pkib	Sigma	For: CTGCTCCCTTAACTGCTGGAT

		Rev: GATTGTGGAAAAGCGTGTGGT
Rgs4	Sigma	For: GAGTGCAAAGGACATGAAACATC<br>Rev: TTTTCCAACGATTCAGCCCAT
Ucn3	Sigma	For: AAGCCTCTCCCACAAGTTCTA<br>Rev: GAGGTGCGTTTGGTTGTCATC
Stx1a	Sigma	For: AAGATTGCCGAAAACGTGGAG<br>Rev: TGCTCAATGCTCTTTAGCTTGG
Snap25	Sigma	For: CAACTGGAACGCATTGAGGAA<br>Rev: GGCCACTACTCCATCCTGATTAT
Vamp2	Sigma	For: GCTGGATGACCGTGCAGAT<br>Rev: GATGGCGCAGATCACTCCC
PPIA	Sigma	For: AAGACTGAGTGGTTGGATGG<br>Rev: ATGGTGATCTTCTTGCTGGT
GJD2	Sigma	For: ATCGGGAGGATCCTGTTGAC<br>Rev: GAGTAGGTGATGAAGCAAAGACTG
PDX1	Sigma	For: TGCTAGAGCTGGAGAAGGAG<br>Rev: TTGATGTGTCTCTCGGTCAA
CACNA1G	Sigma	For: GCTTCGGAACCGATGCTTC<br>Rev: TCCTCGTTCTCTGTCTGGTAAT
CACNA1H	Sigma	For: TCTTCTTCTGCCTCGGTCA<br>Rev: CACGCAGTTGAGCATGATT
CACNA1I	Sigma	For: GGAGCTGATCCTCATGTCCC<br>Rev: CACGGGTTGCACACCATCT
CACNA1A	Sigma	For: GATTTTAGCCACCATCATAGCGA<br>Rev: CCAGCCGTTCAGACATCGG
CACNA1C	Sigma	For: TCCAGAAGATGATTCCAACG<br>Rev: ATTGGGGTGAAAGAGGAGTC
CACNA1D	Sigma	For: CGCGAACGAGGCAAACTATG<br>Rev: TTGGAGCTATTCGGCTGAGAA
CACNA1A	Sigma	For: GATTTTAGCCACCATCATAGCGA<br>Rev: CCAGCCGTTCAGACATCGG
SCN1B	Sigma	For: GTCTACCGCCTGCTCTTCTTC<br>Rev: TGGATGCCATGTCTCTGTTG
SCN3A	Sigma	For: TTCACTAATGCCTGGTGCTG<br>Rev: CCGAGTTCTGAGTAGCCAAGAG
SCN3B	Sigma	For: ATTGTTTCCCCTGGCTTCTC<br>Rev: AGGGCACTTCCACACACAC
SCN8A	Sigma	For: AGCACCATCCTATGACACCAC<br>Rev: TGGCTATGAGCTTCAGGAAC
SCN9A	Sigma	For: TCATCTTTGGGTCATTCTTCAC<br>Rev: ACCCCAGCTTTTTCATTGC
**TaqMan™ probes**		
Gapdh	Fisher Scientific	Mm99999915_g1
Gjd2	Fisher Scientific	Mm00439121_m1
PPIA	Fisher Scientific	Hs03045993_gH
MAFA	Fisher Scientific	Hs04419862_g1
NEUROG3	Fisher Scientific	Hs00360700_g1

Supplementary Table 2: Primer sequences and probe IDs.
