# Supplementary material for: PDX1LOW MAFALOW β-cells contribute to islet function and insulin release
Source: Nat Commun. 2021 Jan 29;12:674. doi: 10.1038/s41467-020-20632-z (PMC7846747; doi:10.1038/s41467-020-20632-z)
Supplement: Supplementary file 3 — Description of Additional Supplementary Files [file 41467_2020_20632_MOESM3_ESM.pdf]

Description of Additional Supplementary Files

File Name: Supplementary Movie 1

Description: Fast Ca2+ recordings in B-NORM islets showing highly coordinated oscillations in response to 11 mM glucose (playback = 70 frames per second).

File Name: Supplementary Movie 2

Description: Fast Ca2+ recordings in B-MAT islets showing more stochastic responses to 11 mM glucose (playback = 70 frames per second).

File Name: Supplementary Movie 3

Description: Ca2+ activity in hM4Di-/- islets before and after application of J60. Application of J60 does not affect Ca2+ oscillations in response to 11 mM glucose in hM4Di-/- islets (playback = 30 frames per second).

File Name: Supplementary Movie 4

Description: Ca2+ activity in hM4Di+/- islets before and after application of J60. Application of J60 to hM4Di+/- isletssilences Ca2+ oscillations in response to 11 mM glucose but not the depolarizing stimulus KCl (added at the end) (playback = 30 frames per second).

File Name: Supplementary Movie 5

Description: Fast Ca2+ recordings in hM4Di-/- islets showing highly coordinated oscillations in response to 11 mM glucose (playback = 70 frames per second).

File Name: Supplementary Movie 6

Description: Fast Ca2+ recordings in hM4Di+/- islets showing more stochastic responses to 11 mM glucose (playback = 70 frames per second).
